# Comprehensive Analysis of Methylome and Transcriptome to Identify Potential Genes Regulating Porcine Testis Development

**DOI:** 10.3390/ijms25169105

**Published:** 2024-08-22

**Authors:** Yue Feng, Yu Zhang, Junjing Wu, Mu Qiao, Jiawei Zhou, Zhong Xu, Zipeng Li, Hua Sun, Xianwen Peng, Shuqi Mei

**Affiliations:** 1 Hubei Key Laboratory of Animal Embryo and Molecular Breeding, Institute of Animal Husbandry and Veterinary, Hubei Academy of Agricultural Sciences, Wuhan 430064, China; fengyue@hbaas.com (Y.F.); zhangyu@hbaas.com (Y.Z.); wujujing@hbaas.com (J.W.); qiaomu@hbaas.com (M.Q.); zhoujiawei@hbaas.com (J.Z.); xuzhong@hbaas.com (Z.X.); lizipeng@hbaas.com (Z.L.); sunhua@hbaas.com (H.S.); 2 Hubei Hongshan Laboratory, Wuhan 430070, China

**Keywords:** DNA methylation, porcine, testicular development, WGBS, RNA-Seq

## Abstract

DNA methylation plays a critical role in regulating gene expression during testicular development. However, few studies report on candidate genes related to the DNA methylation regulation of porcine testicular development. This study examined the differentially expressed genes (DEGs) and their methylation levels in testicular tissues from pigs at 60 days of age (60 d) and 180 days of age (180 d) using RNA-Seq and whole genome bisulfite sequencing (WGBS). It was determined that DNA methylation primarily occurs in the cytosine–guanine (CG) context, and the analysis identified 106,282 differentially methylated regions (DMRs) corresponding to 12,385 differentially methylated genes (DMGs). Further integrated analysis of RNA-Seq and WGBS data revealed 1083 DMGs negatively correlated with the expression of DEGs. GO analysis showed that these genes were significantly enriched in spermatogenesis, germ cell development, and spermatid differentiation. The screening of enriched genes revealed that hyper-methylation repressed *ADAM30*, *ADAM3A*, *DPY19L2*, *H2BC1*, *MAK*, *RPL10L*, *SPATA16*, and *YBX2*, while hypo-methylation elevated *CACNA1I*, *CADM1*, *CTNNB1*, *JAM2*, and *PAFAH1B3* expression. Additionally, the methylation status of the key genes *ADAM3A*, *ADAM30*, *YBX2*, *JAM2*, *PAFAH1B3*, and *CTNNB1* was detected by bisulfite sequencing PCR (BSP). This study offers insights into the epigenetic regulation mechanisms underlying porcine testicular development.

## 1. Introduction

The testis is a highly complex organ with remarkable cellular heterogeneity, consisting of germ cells and somatic cells [1]. The main functions of testicular tissue are producing sperm and secreting androgens [2], and normal testicular development is vital for maintaining spermatogenesis. Spermatogenesis is a methodical process, with different stages of sperm appearing in an orderly fashion. In pigs, round sperm cells appear at 60 days and mature sperm are present at 180 days of age [3]. Pigs, as significant economic and model animals, hold substantial research importance. The development of boar testes influences the onset of sexual maturity and semen quality, which in turn affects reproductive performance. And reproductive performance is an important economic indicator in pig farming [4]. In this research, testicular tissues from two key stages, i.e., from 60-day-old (60 d) boars in the pre-sexual maturity stage and from 180-day-old (180 d) boars that were mature and producing sperm, were selected for the investigation of testicular development [5].

DNA methylation, a key aspect of epigenetics and epigenomics, establishes and maintains the epigenetic state of the genome [6,7]. It plays a critical role in genomic imprinting [8], X-chromosome inactivation [9], disease onset [10], cellular differentiation [11], and tissue development [12]. Whole genome bisulfite sequencing (WGBS) technology allows for the precise identification of DNA methylation patterns across the genome at the single-base level [13], shedding light on its role in testicular development and cell differentiation. Studying DNA methylation changes in the testis across different stages and states enhances our understanding of its relationship with male reproduction. For instance, DNA methylation affects gene expression during testicular development, crucial for maintaining spermatogonia [14], spermatocytes [15], and Sertoli cells [16]. It also plays a pivotal role in spermatozoa formation and ensures proper embryo development post-fertilization by reprogramming gene expression during spermatogenesis [17,18]. DNA methylation changes are linked to several testicular diseases, and these abnormalities can cause infertility and birth defects [19,20]. Furthermore, DNA methylation occurs in three DNA sequence contexts: mCG, mCHG, and mCHH, where H represents A, C, or T.

The integrated analysis of WGBS and RNA-Seq data offers a comprehensive view of how DNA methylation relates to gene expression, enabling the identification of key genes influenced by methylation. For example, the analysis of both WGBS and RNA-Seq in mouse testicular tissues revealed genes and pathways linked to steroid production and spermatogenesis. They indicated how fine particulate matter (PM2.5) may disrupt male reproductive functions through methylation [21]. An analysis of WGBS and RNA-Seq in embryonic velvet goat skin samples (embryonic day 65 and embryonic day 120 velvet goats) identified genes and mechanisms critical to early follicle development [22]. However, the impact of DNA methylation on gene expression during porcine testis development remains unclear. Clarifying this impact is essential for understanding gene expression changes in male reproductive development.

Testicular development involves multiple biological events, with DNA methylation as a key epigenetic mechanism. This modification is essential for gene expression modulation and cell differentiation, influencing normal testicular development and function [23,24]. To further explore DNA methylation regulation during porcine testis development, a comprehensive analysis of WGBS and RNA-Seq was performed. This analysis screened key candidate differentially methylated genes (DMGs) and their enriched signaling pathways. The study aims to provide significant insights into DNA methylation exploration and its potential regulatory mechanisms, facilitating a deeper understanding of male reproductive system biology. It also offers new perspectives for future reproductive health research and therapy.

## 2. Results

### 2.1. DNA Methylation Pattern in Porcine Testicular Tissue

**Figure 1:**
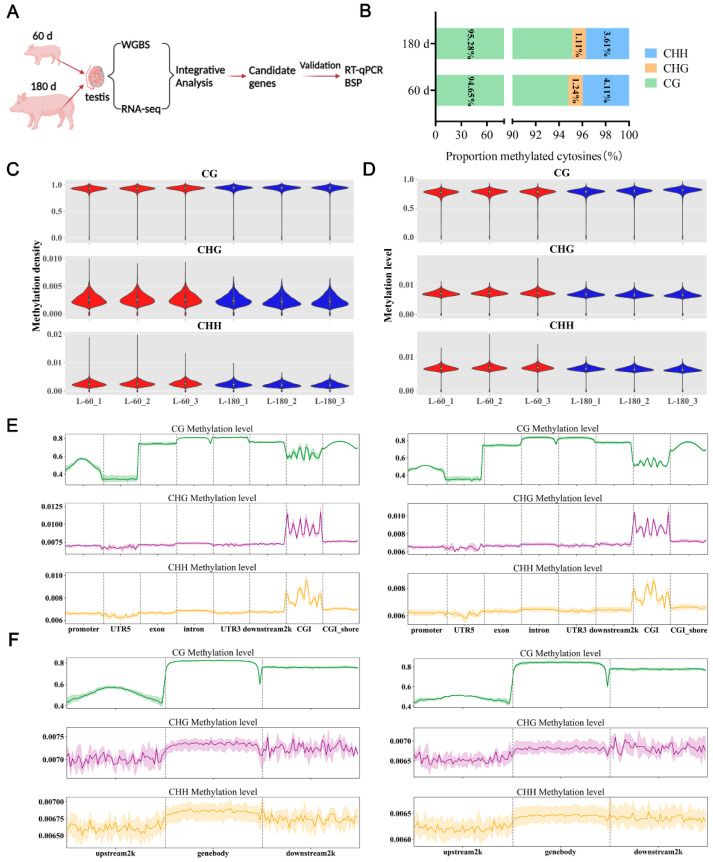
Characterization of DNA methylation in porcine testicular tissue. (**A**) Schematic representation of the experimental design. (**B**) The stacked bar charts shows the average proportion of three distinct methylation types in 60 d and 180 d porcine testicular tissues. The methylation types mCG, mCHG, and mCHH (H = A, C, or T) are represented in green, orange, and blue, respectively. (**C**) Violin plots depict the methylation density in three different sequence contexts (included CG, CHG, and CHH; H = A, C, or T). Each row indicates a distinct context. The *Y*-axes denote the methylation densities, and the *X*-axes represent distinct samples (60 d and 180 d). (**D**) Violin plots illustrate the distribution of methylation levels in three distinct sequence contexts (included CG, CHG, and CHH; H = A, C, or T). Each row indicates a distinct context. The *Y*-axes denote the methylation level, and the *X*-axes represent distinct samples (60 d and 180 d). (**E**) Methylation levels of various genomic elements in 60 d versus 180 d testis tissues. Each row indicates a distinct context. The *X*-axis denotes the distinct genomic elements, and *Y*-axis denotes the methylation level. The left and right panels show the distribution of methylation levels in 60 d and 180 d testis tissues, respectively. The green, purple and yellow lines represent the methylation levels of CG, CHG and CHH, respectively. (**F**) Distribution of methylation levels in upstream/downstream 2K regions in 60 d versus 180 d testis tissues. Each row indicates a distinct context. The *X*-axis denotes various regions, and the *Y*-axis reflects the methylation levels. The left and right panels show the distribution of methylation levels in 60 d and 180 d testis tissues, respectively. The green, purple and yellow lines represent the methylation levels of CG, CHG and CHH, respectively.

Porcine testicular tissues were collected at 60 d and 180 d for WGBS and RNA-Seq analyses. Integrated analysis of WGBS and RNA-Seq data screened potentially relevant genes regulating testicular development; these were validated by RT-qPCR and BSP (Figure 1A). Using Bsmap software (v2.9.0), methylation data comparison and site identification showed that DNA methylation percentages for mCG, mCHH, and mCHG (three different types of cytosine methylation) at 60 d were 94.65%, 4.11%, and 1.24%, respectively (Figure 1B). In contrast, at 180 d, these percentages were 95.28%, 3.61%, and 1.11%, respectively (Figure 1B). A comprehensive analysis of genome-wide methylation densities and levels in 60 d and 180 d testicular tissues was conducted. The comparative analysis revealed significant differences in methylation density and levels of both CHG and CHH contexts in 60 d and 180 d testicular tissues, but not in the CG context (Figure 1C,D).

The analysis of DNA methylation levels across different genomic functional elements at 60 d and 180 d revealed varying methylation levels in the CG, CHG, and CHH contexts. In the CG context, exon, intron, 3′UTR, downstream 2K, and CGI shore exhibited the highest methylation levels, with the promoter region and CGI showing intermediate levels, and the 5′ UTR displaying the lowest level (Figure 1E). Under CHG and CHH contexts, the CGI region displayed high methylation levels, while the other elements showed lower levels (Figure 1E). In the distribution of methylation levels for the upstream/downstream 2K regions in the CG context, the gene body and downstream 2K regions exhibited the highest levels, followed by the upstream 2K (Figure 1F).

### 2.2. Identification of DMRs

**Figure 2:**
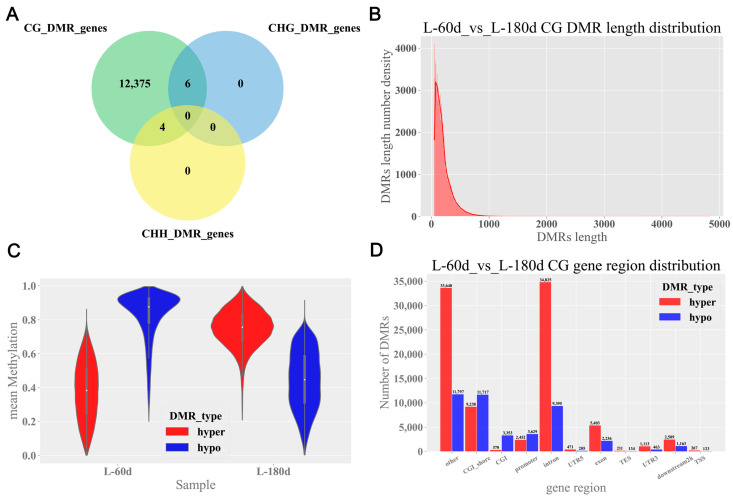
Identification of DMRs in porcine testicular tissue. (**A**) Venn diagram of CG, CHG, and CHH DMR-associated genes. (**B**) The length distribution of DMRs in CG context. The *X*-axis indicates the DMRs length; the *Y*-axis indicates the density at each length, and the DMRs length distribution of fitted curves is marked in red. (**C**) Violin plots of the distribution of methylation levels in the CG context. The *X*-axis represents the different age groups (60 d and 180 d), and the *Y*-axis represents the value of the mean methylation level. (**D**) DMR-anchored regions in CG context. The *X*- and *Y*-axes denote the different gene regions and the number of DMRs, respectively.

DMRs were analyzed against the porcine reference genome (Sscrofa 11.1). A total of 12,385 DMGs were identified (Figure 2A). Among these, 12,385 DMGs were present in the CG context, while 4 genes were identified in the CHH context and 6 genes in the CHG context (Figure 2A). In the CG context, the DMRs mostly ranged from 50 to 200 bp (Figure 2B). The distribution of methylation levels within the DMRs showed that in 60 d testicular tissue, the proportion of hypo-methylated regions was higher than that of hyper-methylated regions. Conversely, in 180 d testicular tissue, the proportion of hyper-methylated regions was higher than that of hypo-methylated regions (Figure 2C). Disregarding other genomic and CGI shore regions, the intron regions contained the most DMRs, followed by the exon and promoter regions (Figure 2D).

### 2.3. Functional Enrichment Analysis of DMGs

**Figure 3:**
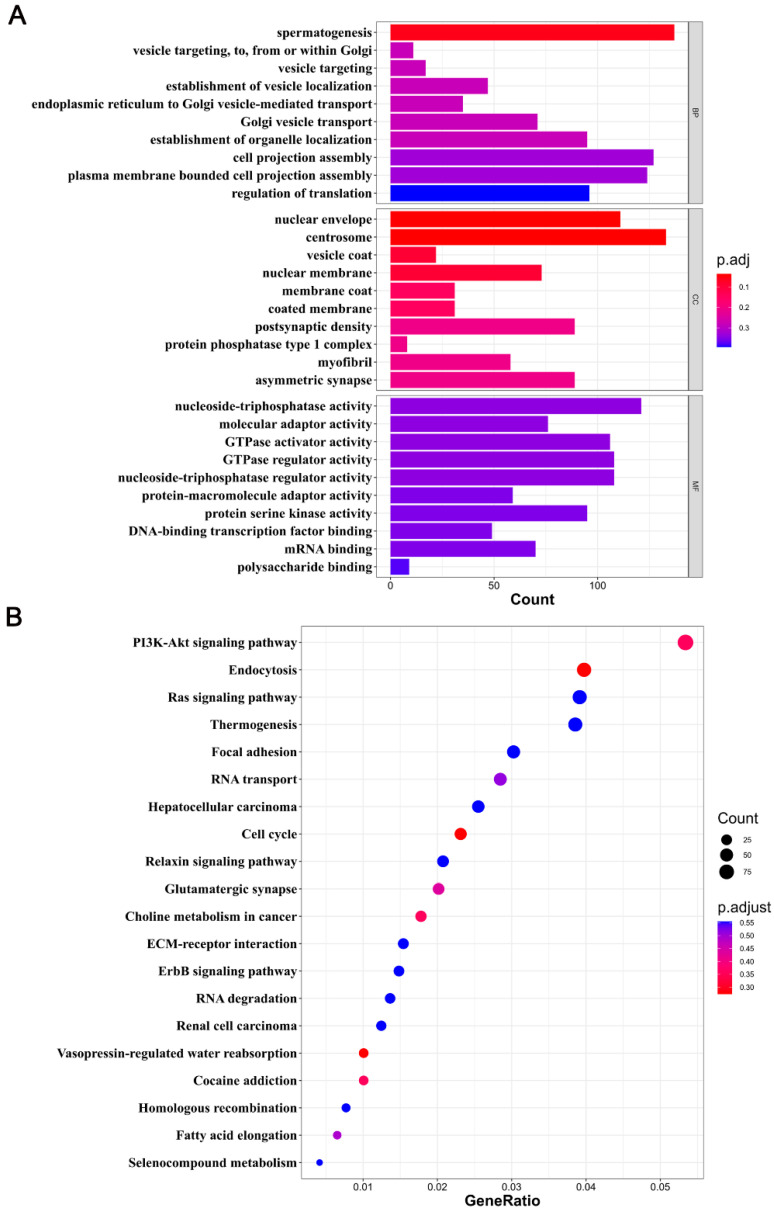
GO and KEGG analyses of DMGs. (**A**) A bar plot shows the GO enrichment analysis in the CG context. The *Y*-axis delineates different GO terms, while the *X*-axis shows the number of DMGs. BP, CC, and MF denote biological processes, cellular components, and molecular functions, respectively. (**B**) Scatter plot illustration of KEGG pathway enrichment in the CG context. The *Y*- and *X*-axes display the pathway names and the gene ratio, respectively. The dot size represents the number of DMGs, while the dot color corresponds to distinct q-values.

To elucidate the functions of genes in DMRs, we annotated 12,385 DMGs using GO and KEGG databases. Since DMGs were primarily found in the CG context, we focused on CG methylation. GO enrichment analyses indicated enrichment in spermatogenesis, nuclear envelope, centrosome, and nuclear membrane processes. Spermatogenesis was notably related to testis development (Figure 3A). Furthermore, KEGG pathway analysis showed that DMGs were mainly enriched in the PI3K-Akt signaling pathway, endocytosis, the cell cycle, and the Ras signaling pathway (Figure 3B). These results highlight the critical role of DNA methylation in regulating spermatogenesis, the cell cycle, and signaling pathways during testicular development.

### 2.4. Association Analysis of DNA Methylation with Differentially Expressed Genes (DEGs)

**Figure 4:**
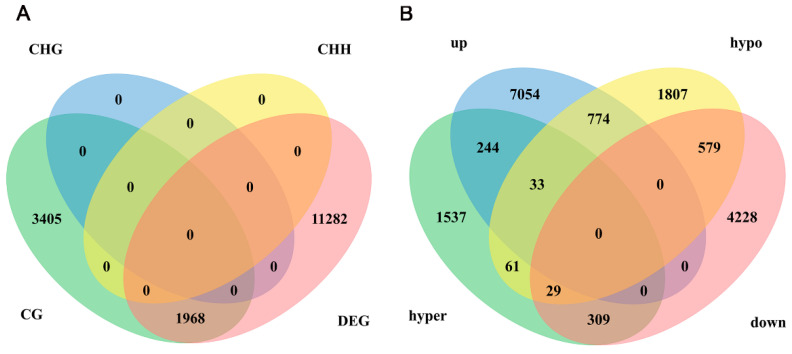
Correlation analysis between DNA methylation and gene expression. (**A**) A Venn diagram illustrates the overlap between DEGs and DMGs (CG, CHG, and CHH contexts). (**B**) The Venn diagram focused on the comparison of DMGs in the CG context with DEGs, differentiating between genes associated with hyper-methylation (hyper) and hypo-methylation (hypo), as well as those with elevated (up) and reduced (down) expression levels.

Transcriptome sequencing of 60 d and 180 d testicular tissue screened out 13,250 DEGs. Correlation analyses were applied to DMGs and DEGs, revealing a significant overlap of 1968 genes in the CG context (Figure 4A). DMGs in the CG context were classified as hyper-methylated and hypo-methylated genes. Among them, there were 2213 hyper-methylated genes and 3283 hypo-methylated genes. Additionally, DEGs were categorized as highly expressed and lowly expressed genes. Among them, there were 8105 highly expressed genes and 5145 lowly expressed genes. Notably, the most significant fraction of DMGs showed a negative correlation with DEGs. Of these, 338 genes displayed upregulated methylation and downregulated gene expression. In contrast, 807 genes exhibited downregulated methylation and upregulated gene expression (Figure 4B).

### 2.5. Enrichment of Key Pathways of DMGs Negatively Associated with DEGs

**Figure 5:**
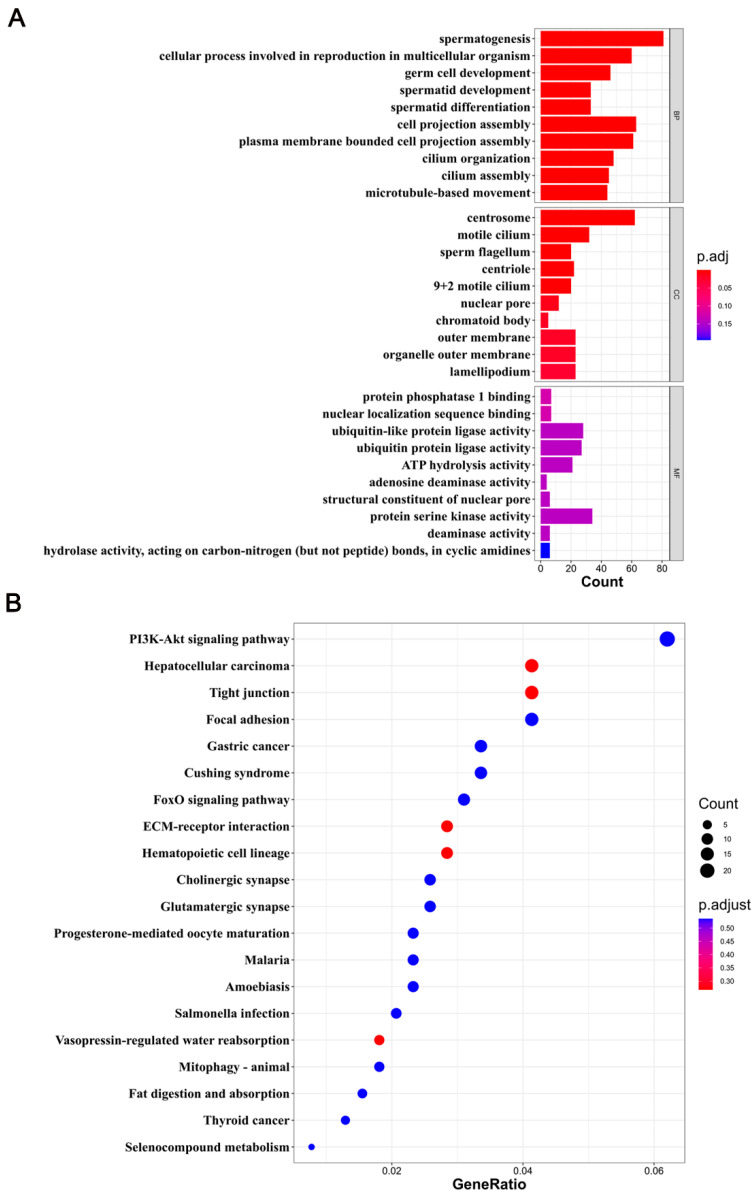
Functional enrichment analysis of DMGs negatively associated with DEGs. (**A**) A bar plot graph shows the GO enrichment of DMGs negatively associated with DEGs. The *Y*-axis denotes different GO terms, while the *X*-axis shows the number of DMGs negatively associated with DEGs. BP, CC, and MF denote biological processes, cellular components, and molecular functions, respectively. (**B**) A scatter plot illustration of the KEGG pathway for DMGs negatively associated with DEGs. The Y and X axes display the pathway names and the gene ratio, respectively. The dot size represents the number of DMGs negatively associated with DEGs in the pathway, while the dot color corresponds to distinct q-values.

A functional enrichment analysis of DMGs negatively associated with DEGs showed significant enrichment of GO functions in the CG context in spermatogenesis, germ cell development, spermatid development, spermatid differentiation, sperm flagellum, and 9+2 motile cilium (Figure 5A). The top 20 KEGG enrichment pathways included those for hepatocellular carcinoma, tight junction, ECM-receptor interaction, hematopoietic cell lineage, and other signaling pathways (Figure 5B). The screening of enriched genes identified *ADAM30*, *ADAM3A*, *DPY19L2*, *H2BC1*, *MAK*, *RPL10L*, *SPATA16*, *YBX2*, *CACNA1I*, *CADM1*, *CTNNB1*, *JAM2*, and *PAFAH1B3* as strongly linked to testicular development and spermatogenesis in pigs (Table 1).

### 2.6. Identification of Genes Involved in Testicular Development

**Figure 6:**
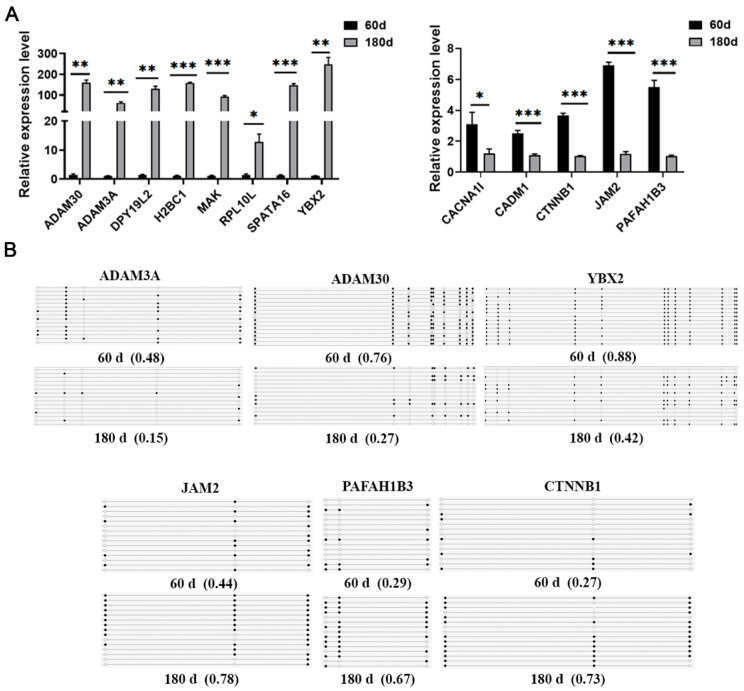
Validation of DMGs and their expression patterns. (**A**) The RT-qPCR expression levels of genes were consistent with RNA-Seq data; hyper-methylation suppressed gene expression and hypo-methylation stimulated gene expression. (**B**) DNA methylation detected by BSP was consistent with WGBS data. Triplicate biological assays were conducted, with *β-actin* serving as a control housekeeping gene. * *p* < 0.05; ** *p* < 0.01; *** *p* < 0.001.

The expression levels of screened genes were verified using RT-qPCR, revealing significant differences between 60 d and 180 d pig testis tissues. Among them, relative expression of *ADAM30*, *ADAM3A*, *DPY19L2*, *H2BC1*, *MAK*, *RPL10L*, *SPATA16*, and *YBX2* increased significantly (*p* < 0.05) in 180 d tissues (Figure 6A), while relative expression of *CACNA1I*, *CADM1*, *CTNNB1*, *JAM2*, and *PAFAH1B3* was significantly reduced (*p* < 0.05) (Figure 6A). Further, to validate the reliability of the WGBS data, BSP was performed on these DMGs. The results confirmed that the methylation trends identified by BSP were consistent with those from WGBS (Figure 6B). This consistency confirms the reliability of WGBS data for further study.

## 3. Discussion

The testicular development of boars is crucial for enhancing productivity in pig farming, with early development largely reliant on spermatogenesis. Therefore, optimizing testicular development in boars will significantly boost the economic efficiency of pig farming. DNA methylation serves as a fundamental regulator in animal development and gene expression [25,26,27]. Although DNA methylation is extensively documented in mammals, its dynamics in porcine testicular development at various stages remain less understood. In this research, we explored the dynamic regulation of DNA methylation and its association with the transcriptome during porcine testicular development, employing WGBS and RNA-Seq methodologies.

DNA methylation is a key epigenetic modification across many animal genomes, crucially influencing growth and development [28,29]. This modification undergoes dynamic regulation through the interaction of methylation and demethylation activities. In animal genomes, DNA methylation predominantly targets cytosine at the CG sites, in contrast to its behavior of this process in plant genomes, where it also includes CHG and CHH sites [30]. Comparative genome-wide methylation patterns in porcine testicular tissues at 60 days and 180 days showed similarities in functional genomic regions. However, there were differences between the CG context and the CHH and CHG contexts, potentially due to sequence differences between different genetic elements [31]. Methylation was the most abundant in the CG context, followed by the CHH and CHG contexts, in 60 d testis tissue (CG: 94.65%, CHH: 4.11%, CHG: 1.24%) and 180 d testis tissue (CG: 95.28%, CHH: 3.61%, CHG: 1.11%). A similar pattern was observed in sheep skeletal muscle and porcine ovary tissues. However, the methylation rates in sheep skeletal muscle and porcine ovary tissues were higher than those in porcine testis tissues, while the rates in regards to the CG contexts were lower than those in porcine testis tissues [32,33]. This underscores the significant role that CG methylation may play in the development of the testis. In porcine testis tissues, the gene body showed high methylation, and loci near transcription start sites (TSS) showed low methylation levels (Figure 1F). Thus, the methylation patterns across CG, CHG, and CHH contexts, along with their biological implications, varied between species and even within distinct tissues of the same species. CG methylation was efficiently maintained in porcine testicular tissues.

GO analysis revealed that DMGs negatively associated with DEGs were mainly enriched in spermatogenesis, germ cell development, sperm flagellum, and spermatid development and differentiation. These processes are critical in testicular development and warrant further analysis. Among them, spermatogenesis is a key process in testicular development, and the enriched genes include the *DPY19L2*, *SYCP3*, *BAG6*, *SPATA16*, *ODF2*, *ADAM3A*, *YBX2*, *ADAM30*, *JAM2*, and *CTNNB1* genes. Research has shown that these genes play roles in spermatogenesis and testicular development. For instance, the *ADAM3A* and *ADAM30* genes display high methylation levels and low gene expression at 60 days, with converse methylation levels and gene expression at 180 days. The literature reports that *ADAM* family members are highly expressed in elongated spermatids and are involved in the sperm–egg binding process [34]. We speculate that this may be due to the reduction of methylation levels in *ADAM* family members during spermatogenesis. *JAM2* and *CTNNB1* genes exhibit low methylation levels and high gene expression at 60 days and high methylation levels and low gene expression at 180 days. *JAM2* and *CTNNB1* genes are highly expressed in Sertoli cells and participate in the interaction between Sertoli cells and other Sertoli cells, or between Sertoli cells and sperm [35,36]. We conjecture that this may be due to the low methylation levels of the *JAM2* and *CTNNB1* genes during early spermatogenesis, which leads to their high expression in the Sertoli cells. The dynamic methylation levels of these genes regulate gene expression, thereby strictly regulating the spermatogenesis process and endowing sperm with fertilization ability. Furthermore, the deletion of dpy-19-like 2 (*DPY19L2*) in mice causes defects in acrosome development and nuclear compaction during sperm formation, leading to male sterility [37]; spermatogenesis-associated 16 (*SPATA16*) is linked to sperm morphology, sperm count, and viability, playing a crucial role in human reproductive health [38]; in mice, Y-box binding protein 2 (*YBX2*) is highly expressed in post-meiotic male germ cells and participates in spermatogenesis [39]; the knockdown of synaptonemal complex protein 3 (*SYCP3*) in mouse testicular tissues results in severely impaired spermatogenesis [40]; the knockout of exon 24 of the mouse Bcl-2-associated athanogen-6 (*BAG6*) gene damages spermatogenesis and reduces male fertility [41]; the deletion of outer dense fiber protein 2 (*ODF2*) in mice leads to abnormal sperm tail results [42].

KEGG analysis indicated that DMGs negatively associated with DEGs were mainly enriched in signaling pathways such as the PI3K-AKT signaling pathway, hepatocellular carcinoma, ECM-receptor interaction, tight junction, and the FoxO signaling pathway. Among these, tight junctions [43], ECM-receptor interactions [44], and the PI3K-AKT signaling pathway [45] are closely related to spermatogenesis, suggesting that DMGs are involved in the dynamic processes of testicular development. As 60-day-old boars are in the pre-sexual maturity stage, their testes are beginning to grow and develop, while 180-day-old boars, which are entering sexual maturity, possess mature testes, and spermatogenesis has begun to function. Therefore, it is speculated that DMGs affect testicular development and spermatogenesis through DNA methylation.

Although the current study offers valuable insights into the regulatory mechanisms underlying porcine testis development, it has limitations. For example, this study relies on a limited sample size and specific developmental stages. Future studies should employ larger sample sizes and a wider range of developmental stages to validate these findings and to further explore the regulatory mechanisms. Additionally, this study revealed a relationship between the methylome and the transcriptome, suggesting that DNA methylation may regulate gene expression through several mechanisms. Future studies are needed to further elucidate these mechanisms and their impact on testicular development through DNA methylation.

## 4. Materials and Methods

### 4.1. Experimental Animals and Sample Collection

The 60 d and 180 d Landrace boars were sourced from a pig breeding farm in Tongren, Guizhou Province, China. They were raised in the same environment, with the same genetic background, similar body weight, and no semen collection behavior. Institutional Review Board Statement: All experimental procedures employed in this study complied with the guidelines set by the Animal Care and Use Committee of Hubei Academy of Agricultural Sciences (HBAAS-2023-025). Testicular tissues from 60 d and 180 d boars (*n* = 3 in each group) were collected under the same conditions. These tissues were immediately frozen at −80 °C for later analysis.

### 4.2. Whole Genome Bisulfite Sequencing (WGBS)

Genomic DNA was extracted using the animal genomic DNA kit from Tiangen, Beijing, China. After extraction, the DNA was fragmented and purified using a PCR purification kit to maintain DNA fragment integrity and quality. Fragmented DNA samples were end-repaired, received an “A” nucleotide at the 3′ end, and were ligated with methylated adapters. After ligation, DNA fragments underwent bisulfite conversion with the Methylation-Gold kit from ZYMO, USA. High-quality reads, free from low-quality scores and unknown nucleotides, were prepared for downstream analysis. These reads were then aligned to the porcine reference genome using BSMAP software (v2.9.0) [46].

### 4.3. Bisulfite Sequencing PCR (BSP)

BSP was used to identify the methylation status of DNA within selected candidate genes. Genomic DNA was transformed using sodium bisulfite with the EZ DNA Methylation-Gold™ Kit (Zymo Research, Irvine, CA, USA). The bisulfite-converted genomic DNA was then amplified by PCR using Zymo Taq™ DNA polymerase (Zymo Research, Irvine, CA, USA). After amplification, the PCR products were purified using a Gel Extraction Kit (Qingke Biotechnology, Wuhan, China), ligated, and cloned into the pMD19-T vector (TaKaRa, Shiga, Japan). A total of 15 clones per sample were randomly selected for DNA sequencing analysis. Primers for the genes used are listed in Table 2.

### 4.4. RNA-Seq

Total RNA was extracted from testicular tissue using TRIzol reagent from Invitrogen. RNA quality and integrity were assessed using a NanoDrop 2000 (Thermo Fisher Scientific, Waltham, MA, USA) and a LabChip GX microfluidic capillary electrophoresis instrument (PerkinElmer, Waltham, MA, USA). RNA-Seq analysis was performed on an Illumina platform and processed by Annoroad Gene Technology in Beijing, China. The library quality was verified, and libraries were assembled based on concentration and data volume targets. CASAVA [47] converted high-throughput sequencer images into analyzable reads. Clean reads were mapped to the porcine reference genome using HISAT2 v2.0.5 [48]. Differential expression analysis was conducted using DESeq2 (v3.17) [49].

### 4.5. Reverse Transcription qPCR (RT-qPCR)

Total RNA was extracted from testicular tissue using TRIzol reagent (Invitrogen, Carlsbad, CA, USA). The RNA was washed twice with 75% ethanol. Genomic DNA was removed using the gDNA eraser kit (Takara, Shiga, Japan). RNA was reverse-transcribed into cDNA using a reverse transcription kit (Takara, Shiga, Japan). RT-qPCR analysis was conducted using SYBR Green Supermix (Bio-Rad, Hercules, CA, USA). Each gene in each sample was assessed three times. The 2^−∆∆Ct^ method was used to calculate relative mRNA levels. *β-actin* served as a control housekeeping gene. The RT-qPCR primer sequences are listed in Table 3.

### 4.6. Data Analysis

The fastp program, v.0.23.4 [47], was utilized to conduct quality control and to filter and trim the raw sequencing data. MethylDackel (v0.6.1) was used to extract all CpG sites and read counts in a format suitable for the R analysis package methylkit [50]. Differentially methylated regions (DMRs) were identified using metilene v0.2-8 [51]. Finally, GO enrichment and KEGG pathway analyses were conducted using ClusterProfiler v4.0 [52].

### 4.7. Identification of DMRs and Functional Analysis of DMR-Associated Genes

According to the distance between C sites, C sites within a certain range are classified into a region to be detected, and then combined using double statistical tests (MWU-test and 2D KS-test), whereby the DMR retesting of pairs of samples or two groups of samples can be quickly realized during this period. Finally, differential methylation regions were obtained through multiple testing correction. These regions were further classified as differentially methylated genes (DMGs), if they were located within the gene body or 2 kb upstream or downstream of it. To elucidate the functions of DMGs, GO enrichment and KEGG pathway analyses were performed.

### 4.8. Statistical Analysis

To ensure result reliability, at least three independent experiments were performed and analyzed. Data are presented as mean ± SD. A two-tailed Student’s *t*-test was used to determine significant differences between groups. Data with * *p* < 0.05 were considered statistically significant.

## 5. Conclusions

This study explored the relationship between DNA methylation and gene expression during porcine testis development. It systematically identified the DMRs involved by integrating DNA methylation and transcriptome data analysis. A total of 13 genes potentially related to this development were identified. The screening of enriched genes showed that hyper-methylation repressed the expression of *ADAM30*, *ADAM3A*, *DPY19L2*, *H2BC1*, *MAK*, *RPL10L*, *SPATA16*, and *YBX2*. In contrast, hypo-methylation elevated *CACNA1I*, *CADM1*, *CTNNB1*, *JAM2*, and *PAFAH1B3* expression. In conclusion, this study provides valuable insights for exploring potential regulatory mechanisms of DNA methylation and porcine testis development. These results can be used for the marker-assisted selection of boar semen characteristics to improve semen quality and the reproductive traits of breeding pigs, thereby improving the reproductive efficiency of the entire pig herd.

## Figures and Tables

**Table 1:** Candidate genes for testicular development were screened from DMGs negatively associated with DEGs.

Gene Name	Log2 Fold Change	Diff. Methy	C Context	Region
*ADAM30*	11.5478768495596	−0.533689	CG	promoter
*ADAM3A*	10.1525323821239	−0.423786	CG	promoter
*RPL10L*	9.31946493147972	−0.52811	CG	promoter
*SPATA16*	9.13952557495772	−0.53494	CG	promoter
*H2BC1*	8.9679817878648	−0.640505	CG	promoter
*DPY19L2*	8.31772645532461	−0.502483	CG	promoter
*YBX2*	8.27754426747838	−0.520806	CG	promoter
*MAK*	7.80066048376145	−0.612207	CG	promoter
*CTNNB1*	−1.20022448264121	0.41598	CG	promoter
*CADM1*	−1.21820498177358	0.415944	CG	promoter
*PAFAH1B3*	−1.3516785322754	0.324475	CG	promoter
*JAM2*	−2.0339454194098	0.280444	CG	promoter
*CACNA1I*	−2.16972157288754	0.234783	CG	promoter

**Table 2:** Primer sequences for amplification of DNA methylation-related genes.

Gene	Sequence of the Primers
*ADAM30*	F: GYGATGTGGGTATAGTGGGT R: ACAAATACACRCCCTATCCAA
*ADAM3A*	F: GGTGATTTAGGTTTTATAAGAGTAT R: TACATATATTCAAATATTTCTTTCC
*YBX2*	F: TTATGTAATTAATATTTTAATTAAGGG R: ATCTCAAACCTCRCTTAATAA
*JAM2*	F: TTTGATTGAAAATAATGTATTAAGTT R: CCTTTCCTTTCCTACTCTTTAA
*PAFAH1B3*	F: TTTATTGAGTATTTATTGTTTGTGTT R: AACTTTTTAAATTTAAACAAACAAC
*CTNNB1*	F: TAGGTGGAAGGGAAGTTAAA R: TTCCCATAATAATAACATTTTAAT

**Table 3:** Primer information for RT-qPCR.

Gene	Sequence of the Primers
*ADAM30*	F: GCGATGGTACTTCCTGTGGT R: CTCGGTGGTTGCACTTCTCA
*ADAM3A*	F: CACAGATCGTACCAAAGACGG R: GTCCATTATCACAAAACCTTCCG
*DPY19L2*	F: GCCTTCTGGTATCGCTCAGT R: GGGTCTCCCAATCCTTCACAG
*H2BC1*	F: CAGAAGAGGCCGGAAAGAGA R: GTGGAACGCTTGCTGTAGTG
*MAK*	F: AGAGTCAACAGAAACAGCCCC R: AGTGCCGTTGGGCTTGATTG
*RPL10L*	F: ATGGTTTCCTTGTGGGGAGATA R: GCAGAAGCGTGACTTTGGATA
*SPATA16*	F: GCTGTGGAGATAAAAAGGTAGAAAT
	R: CTTTGCCTCTTTCTTTCCAG
*YBX2*	F: GTCTTTGTTCACCAGACAGCTA
	R: ACATCGAACTCCACGGTCTC
*CACNA1I*	F: TCACCGTGTTCCAGATCCTCAC
	R: TTCAGAGTAGGAGCGATTGGCG
*CADM1*	F: CAGAATTTGTTTACTAAAGACGTGA
	R: GTCCCTGAAATAAATGGTCTGC
*CTNNB1*	F: ATGGCTACCCAAGCTGATTTGAT
	R: GGTCGTGGCACCAGAATG
*JAM2*	F: CCCTGGAAGTATTAGTGGCTCC
	R: GGGAGCTGGATTGCCTTCTT
*PAFAH1B3*	F: AACACATCCGACCCAAGATTGT
	R: TTCTCACGGAGTGGATTGGG
*β-actin*	F: CCAGGTCATCACCATCGG R: CCGTGTTGGCGTAGAGGT

## Data Availability

The data that support the findings of this study are available from the corresponding author upon reasonable request.
